# A review on the biological effects of nanomaterials on silkworm (*Bombyx mori*)

**DOI:** 10.3762/bjnano.12.15

**Published:** 2021-02-12

**Authors:** Sandra Senyo Fometu, Guohua Wu, Lin Ma, Joan Shine Davids

**Affiliations:** 1 School of Biotechnology and Sericulture Research Institute, Jiangsu University of Science and Technology, Zhenjiang 212018, PR China; 2 School of Environmental and Chemical Engineering, Jiangsu University of Science and Technology, Zhenjiang, 212018, PR China

**Keywords:** biological effects, *Bombyx mori*, nanomaterials, nanotechnology, sericulture

## Abstract

The production of high-quality silkworm silk is of importance in sericulture in addition to the production of biomass, silk proteins, and animal feed. The distinctive properties of nanomaterials have the potential to improve the development of various sectors including medicine, cosmetics, and agriculture. The application of nanotechnology in sericulture not only improves the survival rate of the silkworm, promotes the growth and development of silkworm, but also improves the quality of silk fiber. Despite the positive contributions of nanomaterials, there are a few concerns regarding the safety of their application to the environment, in humans, and in experimental models. Some studies have shown that some nanomaterials exhibit toxicity to tissues and organs of the silkworm, while other nanomaterials exhibit therapeutic properties. This review summarizes some reports on the biological effects of nanomaterials on silkworm and how the application of nanomaterials improves sericulture.

## Introduction

Nanomaterials have unique optical, electronic, and photocatalytic properties which are applied in various fields of science, such as agriculture, cosmetic, food, medicine, and pharmaceutical sectors [1–4]. Despite the positive contributions of nanomaterials, which include the manufacturing of self-cleaning windows, sunscreen, and thickening agents [5–6], the safety regarding their application to the environment and in humans remains a major area of scrutiny.

Model organisms, such as *Arabidopsis Thaliana*, *Xenopus laevis*, *Drosophila melanogaster*, and rats have been used over the past decades to study the safety of nanomaterials on humans and on the environment [7–8]. The use of these model organisms in research has paved the way for new discoveries in science. However, the use of mammalian animal models, such as pigs, mice, and primates have sparked bioethical problems since experimental trials may cause pain and discomfort to the animals. Thus, it is imperative that experiments that involve the use of animal models are performed within an ethical framework [9–10].

Nanotechnology is a relatively new scientific area which enabled significant developments in the fields of agriculture, cosmetics, food, and medicine [11–15]. Among other positive contributions, the addition of nanomaterials to packaging materials and films in the food industry is reported to have curbed issues regarding food spoilage and contamination [16], pests, and disease management due to the usage of a nanomaterial-based formulation in agriculture [17]. The inclusion of nanomaterials in sericulture is new; therefore, it is imperative to exploit their effects on silkworms and on silk regarding the improvement of fecundity, survival rates, management of pests, and disease prevention. This review highlights the impacts of nanomaterials on larval body growth and on the development of silkworms. In addition, it discusses the effects of nanomaterial usage on silk quality as well as the effects caused by nanomaterials on silkworm tissues and organs.

## Review

### Applications of nanomaterials in various sectors

Nanotechnology, which involves the use of nanomaterials with sizes in the range of 1–100 nm [18–19], is considered a new technological revolution in science. In agriculture, nanotechnology has provided solutions for issues related to plant protection, nutrition, and pesticide resistance [20–22]. For example, 250 µg/mL of magnesium dioxide nanoparticles (MgO NPs) has been reported to improve the weight and the height of tobacco plants and it was also successful in reducing the severity of bacterial wilt caused by *Ralstonia solanacearum* when compared to control or to other magnesium dioxide nanoparticle concentrations (50 and 150 µg/mL) [23]. The ability of MgO NPs to generate more reactive oxygen species (ROS) and to induce oxidative stress could be a reason for their antibacterial activity against *R. solanacearum* in tobacco plants [23].

Aside from MgO NPs, other nanomaterials, including titanium dioxide (TiO_2_ NPs), zinc oxide (ZnO NPs), copper oxide (CuO NPs), graphene oxide, silver nanoparticles (Ag NPs) [24–26], quantum dots, and superparamagnetic particles [27] have been reported to have antibacterial properties against *Streptococcus mutans* [28] and *Xanthomonas perforans*, antifungal properties against *Fusarium oxysporum* [27] and *Fusarium graminearum* [29] and antiviral properties [30]. TiO_2_ NPs improved the water and oxygen intake by spinach leaves [31], which led to an increase in the growth and germination rate of the spinach seeds. In addition, a higher percentage of dry weight and chlorophyll formation over the germination period of 30 days were observed [32]. TiO_2_ NPs have also been documented to increase the germination of lettuce seeds [33]. Recent reports indicate that multi-walled carbon nanotubes (MWCNTs) also increased the germination of tomato seeds by increasing the seed water uptake [34]. These reports of nanomaterials improving the germination of seeds prove that the incorporation of nanotechnology in agriculture can boost crop production and the use of nanoscale pesticides [35] can improve crop protection. Silica is an example of a nanomaterial used in the formulation of nanoscale pesticides, which are used to control pest incidence [36] on farms.

As mentioned earlier, nanomaterials are used in various sectors and it is imperative to assess their impact on the environment and on human health. Animal models are extensively used in order to elucidate the mechanisms underlying biological processes, to test the efficacy of new drugs [37] and chemicals, and to evaluate nano–bio interactions. The exposure of nanomaterials (NM) to the environment is reported to have detrimental effects on human lungs. Pulmonary inflammation was reported in C57BL/6N mice exposed to zinc oxide (ZnO) (size of nanoparticles functionalized with triethoxycaprylylsilane: 130 nm, size of non-functionalized nanoparticles: 100 nm). Conversely, mice exposed to 100 mg/mL of Ag NM (<20 nm) exhibited no adverse effects on the lungs [38]. Also, anatase TiO_2_ NM was reported to induce pulmonary inflammation in mice models [39]. Kim et al. reported that male Sprague-Dawley rats exhibited neurotoxic behavior after receiving an intracerebroventricular injection containing 2 mg/mL of TiO_2_ NPs [40].

The release of nanomaterials into the environment was reported to be toxic to living organisms, causing impairment of major neurological functions and death. For example, Liu et al. [41] reported that the dietary uptake of carbon-based nanomaterials (fullerene C60, carbon black, single-walled nanotubes, multi-walled nanotubes) had no toxic effect on the growth and development of *Drosophila melanogaster* (fruit fly) from the egg stage to the adult form. However, the dietary uptake of carbon black and single-walled nanotubes caused impairment in the locomotor function of these flies.

Demir et al. [42] reported that Ag NPs (0.1, 1, 5, and 10 mM) induced genotoxicity in the wing spot assay of fruit flies via somatic recombination. Carmona et al. [43] reported cytotoxic effects in the midgut and imaginal disc tissues following an exposure of the fruit fly larvae to TiO_2_ NPs. TiO_2_ NPs did not influence the wing spot assay of the fruit fly; however, a significant increase in DNA damage was recorded when compared to the DNA damage caused by bulk TiO_2_. Panacek et al. [44] reported that fruit flies exposed to 20 mg/L of Ag NPs were unable to complete their lifecycle. However, after a long-term exposure to Ag NPs, later generations of flies had fecundity levels similar to those of the fruit flies from the control group.

Its natural habitat, rapid life cycle (≈4 days), sensitivity, and moderate body size make *Caenorhabditis elegans* (nematode) [45] an ideal model organism to study environmental nanotoxicity at the nanoscale level. *C. elegans* was used as a model organism to evaluate the impact of an exposure to a 50–100 mg/L dose of graphite, graphite oxide nanoplatelets, and graphene quantum dots on its motor nervous system. The locomotion of the nematodes deteriorated following the exposure to these nanomaterials with damages in the dopaminergic and glutamatergic neurons [46].

**Figure 1:**
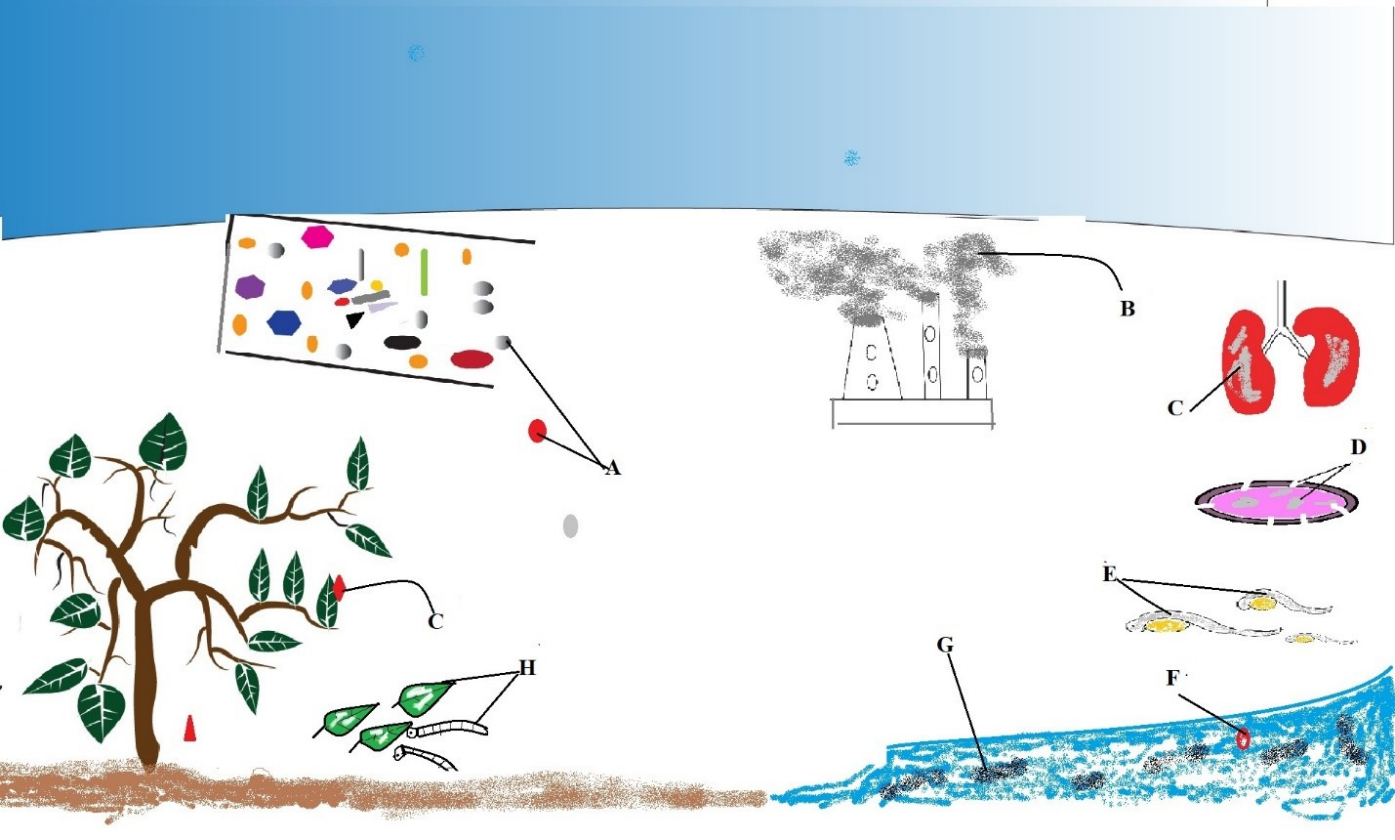
The effects of nanomaterials on the environment and on human and animal health. (A) Nanomaterial waste; (B) fumes and gases from factories; (C) inflammation of the lungs after inhalation of gases/fumes or when in contact with nanomaterial waste; (D) damage in the membrane and in the lumen of silkworm silk glands; (E) dead zebrafish embryos; (F) NM; (G) contamination of water bodies; (H) silkworm feeding on mulberry leaves containing chemical residues.

Fast embryonic development outside the parent zebrafish is an attractive feature that allows for the cell development to be examined from the outside [47]. The exposure of zebrafish to Ag NPs inhibited the hatching rates and caused high embryo mortality. Bar-Ilan et al. [48] reported that colloidal Ag caused a higher mortality of zebrafish embryos when compared to colloidal Au 120 h post-fertilization. Asharani et al. [49] also reported the toxic effects of Ag NPs on zebrafish embryo mortality, delay in hatching, heart rate reduction in the embryo and also non-lethal effects of Au NPs on embryo development. Muller et al. [50] stated that 1.88 μM of dissolved Cu^2+^ inhibited the proteolytic activity of the zebrafish hatching enzyme 1 (ZHE1), which caused a delay in the hatching of zebrafish embryos by 50% following its exposure to copper oxide (CuO). The group of d’Amora [51] compared the toxicity of oxidized carbon nano-onions, oxidized carbon nano-horns, and graphene oxide on the development of zebrafish. Concentration levels above 50 μg/mL of graphene oxide caused a higher mortality rate, delayed the hatching rate, impaired movements, and delayed embryonic development when compared to oxidized carbon nano-horns. These reports indicate that the exposure to some nanomaterials poses a threat to human health and to the environment, as illustrated in Figure 1.

Nanotechnology is in the commercialization exploration phase [52] and it is imperative to explore its benefits. Currently, food industries are interested in exploring the potential benefits of incorporating nanotechnology in food production and food packaging [53]. There is a need to improve food packaging quality to reduce food spoilage caused by pests and also to enhance the shelf life of food products. Nanomaterials are used as an additive in food packaging [54]. Silver nanoparticles, for example, were used as an additive in the manufacture of food containers [55–57] and it was reported that the inclusion of Ag NPs helped to reduce bacterial growth, which prolonged the shelf life of foods stored in those containers. Examples of containers made with Ag NPs include FresherLonger^TM^, Kinetic Go Green, and BlueMoonGoods^TM^ [55–57]. Other uses of nanomaterials include the production of soaps, skin moisturizers, and plastic films as seen in Table 1.

**Table 1:** Selected nanomaterials and their use in different industries.

Nanomaterials	Industry	Uses and effects	References

carbon nanoparticles	cosmetic	anti-aging creams and moisturizers due to their ability to penetrate the skin	[58]
nanoclay composites	food packaging	beverage and food packaging film to prevent oxygen and carbon dioxide penetration from the outside, while retaining food moisture on the inside	[59]
ZnO NPs	food packaging	food and beverage packaging for protection against bacterial contamination	[60–61]
TiO_2_ NPs	food packaging	antifungal activity of TiO_2_-coated polypropylene (PP) plastic film for fruit packaging	[62]
TiO_2_ NPs	agriculture	reduction of pesticide residues in tomato leaves and in the soil by increasing photocatalytic degradation	[63–64]
carbon nanotubes	agriculture	removal of toxicants and contaminants from the water	[65]
silicon dioxide	food	utilized as a thickening agent and also as a beverage fining agent	[66]
TiO_2_ NPs	food	sugar glazing in confectionery	[67]
copper nanoparticles (Cu NPs)	oil and gas	utilized due to their antimicrobial properties and as an additive in lubricants	[68]
zinc nanoparticles (Zn NPs)	oil and gas	used as a catalyst in organic hydrogen reaction and in automobile tail gas disposal	[69]
TiO_2_ NPs	medical	incorporated into orthopedic materials to enhance their applicability in chiropractic activities, tissue engineering, drug and gene delivery	[70–73]
TiO_2_ NPs	food packaging	biodegradable and antimicrobial nanocomposite material made with whey protein for food packaging	[74]

### Silkworm as a model organism

Invertebrate model organisms are the most preferred in experimental trials because of their short generation time and prolific nature when compared to mammalian models, which are costly and involve time-consuming experiments. The rearing conditions of invertebrate model organisms do not require sophisticated infrastructure given their small or moderate body size when compared to mammalian model organisms, which require elaborate laboratory facilities. This could be a reason why scientists are leaning towards the use of invertebrates in experimental trials. In addition, invertebrates are considered excellent models to study defense and behavioral mechanisms [75–77]. Model organisms such as *Drosophila melanogaster*, and *Caenorhabditis elegans* have been used extensively in studying cellular toxicity, response to new drugs [78] and environmental pollution.

Silkworm (*Bombyx mori*) is an invertebrate insect widely used as a model organism in life sciences [79] since it has diverse mutant strains, a complete genome sequenced, and a protein database available [80–81]. There are numerous advantages in using silkworm as a model organism. These animals require less sophisticated infrastructure for rearing, are prolific breeders, have a short life cycle, a moderate body size which allows for an easy manipulation of genes and organs, a clear genetic background, and abundant mutation resources [82–83]. It is relatively easy to obtain organs and genes from the silkworm compared to the fruit fly, which requires the use of a microscope due to its small body size. The fruit fly is also a prolific invertebrate insect with notable features and its genes are used as tools to study embryonic development, organogenesis, neuronal development, aging, inheritance, and cancer [84–85]. Aside from its rapid lifecycle, it is reported that the fruit fly produces genetically identical offspring when compared to mammalian models, which produce a smaller number of offspring at a time [86–87]. Also, the fruit fly has been reported to respond to drugs that act on the central nervous system (CNS) which is similar to the response recorded in mammalian models [88–90].

The similarity between silkworm genes and some human-related genes affected by certain diseases has been studied. Matsumoto and colleagues used silkworm as a model organism in studying the effects of human insulin in hyperglycemic silkworms [91]. Reports from this study indicated that the sugar levels in the silkworm hemolymph reduced after insulin was administered. The group also reported that silkworms fed with a high glucose diet for over 18 h exhibited a hyperlipidemic phenotype and the administration of pioglitazone or metformin improved the glucose tolerance in hyperlipidemic silkworms. The downregulation of the PARK7/DJ-1 gene generated p-translucent (*op*) silkworms by reducing xanthine oxidase synthesis and elevating body oxidative stress response, which makes the p-translucent silkworm a good model to study Parkinson’s disease [92–93].

Mammalian model organisms are mostly used to study the efficacy of new drugs for human-related diseases and also in the screening of antimicrobial drugs [94]. Recent reports indicate similarities between silkworms and *Mus musculus* (mice) regarding their response to antifungals. The median effective dose (ED_50_) and the median lethal dose (LD_50_) of *Candida tropicalis* and of *Candida albicans* were introduced into the silkworm and it was reported that the response of the silkworm to amphotericin B and to fluconazole was consistent with the response observed in a murine model. Fluconazole was efficient in inhibiting the growth of the fungal strains in the silkworm while amphotericin B was most efficient in curbing the fungal growth in the mouse [95]. Ishii et al. focused on studying the function of cytokines in the immune response of insects using the *Bombyx mori* silkworm as a model. It was shown that the activation of a paralytic peptide resulted in cellular and humoral immune responses, which contribute to the host defense in the silkworm *Bombyx mori* [96]. It was also reported that β-glucan, fucoidan, and curldan induced silkworm muscle contraction and induced innate immune responses [97]. This also proves that silkworms can be used as a model to study biological processes, including cell metabolism, gene regulation, and homeostasis [98].

Silkworm is gaining prominence in research as a model organism since it has provided relevant data in various fields and its application does not spark any bioethical issues [81]. Despite the milestones achieved in genomics, the use of silkworms as a model to study mammalian toxicology is limited due to differences in biological processes and organs between these two classes of animals. Hamamoto et al. reported that the absence of neurotoxin receptors in silkworms could be a reason why the levels of neurotoxic substances were higher in silkworms when compared to mice [99]. The silkworm is an invertebrate organism that has a distant relationship with mammals [99], thus, it cannot express or fully mimic the mammalian biological system. This limits their application as a model organism to evaluate the efficacy of drugs in certain toxicology studies. The silkworm as a model organism provides a fresh perspective on how to solve scientific problems and on how to understand some biological processes. Table 2 summarizes the advantages and limitations of using certain model organisms in research.

**Table 2:** The merits and limitations of selected model organisms.

Model organisms	Advantages	Limitations

*Drosophila melanogaster* (fruit fly)	Rapid generation time, highly prolific, low cost, and provides excellent genetic tools to study human-related disorders including cancer and tumor [100–101].	Morphological differences between fruit flies and humans represent a limitation in the use of the former in new drug discovery studies [101]. Due to the small body size of the fruit fly a microscope is required in the experimental trials [102].
*Danio Rerio* (zebrafish)	Zebrafish exhibits complex behaviors and can be used as an animal model to study human behavior. It has a rapid embryonic development and it is used in innate and adaptive immunity studies [103–104].	Difficulties in establishing cell cultures and lack of a conventional knockout technology [104]. Zebrafish cannot be used to study infectious human diseases because bacterial pathogens require a temperature of 37 °C and zebrafish is maintained at 28 °C. This may result in inaccurate data as a low temperature may lead to an attenuated virulence of mammalian infectious agents [104].
*Caenorhabditis elegans* (nematode)	Short generation time, prolific, transgenic strains available, and are self-fertilizing organisms [105–106].	*C. elegans* does not exhibit complex behavior due to the presence of fewer mammalian gene homologs [107]. It has a functioning innate immune system but lacks adaptive immunity [108–109].
*Mus musculus* (mouse)	Used as a model organism in stem cell research [110] and to study human-related diseases including cancer, hypertension, diabetes, osteoporosis, and glaucoma [111].	The housing of mice requires a large infrastructure and this is a problem when the laboratory space is limited. The inability of mice to mimic certain human disease phenotypes, despite the presence of human homolog genes [112], is documented.
*Bombyx mori* (silkworm)	*S*ilkworm has not sparked any controversies and it is considered a safe and economical animal model [99,113]. Complete genomic sequences are available. Organs such as the midgut, silk gland, and fat bodies are easy to be obtained for research purposes [114].	Despite its genetic traits, having a complete genome and protein database available, the silkworm cannot be used as a model organism to study human-related diseases such as neurogenerative disorders and cardiovascular-related complications [115].

### Impact of nanomaterials on the growth and development of silkworms

The feeding efficiency of silkworm larvae is important as it accounts for their growth rate and development [116]. Li et al. [116] showed that low concentrations of nanoparticles (NPs) enhance larval body growth and feeding efficiency. Silkworm larvae fed with 5 or 10 mg/L of TiO_2_ NPs (with sizes in the range of 5–6 nm) improved the ingestion and digestibility of mulberry leaves, which significantly accelerated their body weight gain when compared to the control group and to larvae treated with different concentrations of TiO_2_ NPs (40, 80, and 160 mg/L) after 168 h. During the larval stages, silkworms feed on mulberry leaves which have all the required nutrients needed for their growth [117] and development. The fat body of the silkworm is responsible for the storage, utilization, and transfer of the nutrients required for the growth and development of the silkworm larvae [118]. Tian et al. [119] focused on studying the impact of 5 mg/L of TiO_2_ NPs on the nutrient metabolism of the silkworm fat body. The treatment with TiO_2_ NPs activated the insulin signaling pathway of the silkworm by enhancing the metabolism of carbohydrates, proteins, and fat when compared to the control group. Ni et al. [120] reported that feeding silkworm larvae with 5 g/mL of TiO_2_ NPs led to the development of larger testes and ovaries when compared with the control group. Pandiarajan et al. reported that the exposure of silkworm larvae to 1 ppm of Ag NPs improved the larval growth rate and the cocoon weight when compared to silkworms exposed to 10 and 100 ppm of Ag NPs. It was also indicated that silkworms fed with 100 ppm of Ag NPs exhibited the highest mortality rate when compared to silkworms exposed to other Ag NPs concentrations [121]. Meng et al. reported that silkworm fed with ≥800 mg/L of Ag NPs had a higher mortality when compared with the control group and with groups that received lower concentrations of Ag NPs [122].

Reactive oxygen species (ROS), which are involved in cell signaling and homeostasis [123], are considered a characteristic side-effect of oxygen metabolism. High levels of ROS in living organisms induce oxidative stress, which results in damage to the DNA, proteins, and lipids [124]. This occurs as a result of the imbalance of free radicals in the organism [125]. ROS generation in living organisms is essential as it activates cell defense mechanisms and their antioxidant enzymes [126–127]. Xu et al. [128] reported that silkworm larvae exposed to 10–70 μg/mL of ZnO NPs via subcutaneous injection activated superoxide dismutase (SOD), catalase (CAT), and glutathione peroxidase (GSH-PX) antioxidant enzymes. These enzymes are liable for eliminating excess ROS , and therefore the activation of these enzymes is connected to the expression of apoptosis-related genes within the silkworm midgut (e.g., Dronc, Caspase, and Trt). The research group also indicated that the expression levels of these enzymes within the silkworm midgut were considerably higher within the ZnO NPs exposed group when compared to control and toxicity of ZnO NPs was time-dependent because the antioxidant enzymes remained active 12 h post-injection with a decline in antioxidant activity 36 h post injection.

High concentrations of nanomaterials are detrimental to the organs and tissues of experimental animal models. For example, high concentrations of TiO_2_ NPs were reported to affect the reproduction of CD-1 mice and *Drosophila* [129]. Also, liver damage was reported in mice following treatment with silica nanoparticles for three months [130]. Higher concentrations and sublethal doses of some nanomaterials result in higher mortality, poor cocoon quality, and lower body weight when compared to control groups. Liu et al. [131] exposed 5th-instar silkworm larvae to sublethal doses (0.08 and 0.32 nM) of cadmium telluride quantum dots (CdTe QDs) with sizes of 530 and 720 nm for 48 hours. The larvae exposed to quantum dots exhibited lower larval body mass when compared to the control group and the mortality rates were significantly higher in the QD 530 nm group. They demonstrated that CdTe QDs with a particle size of 530 nm induced higher hemocyte apoptosis when compared to the CdTe QDs 720 nm group due to their smaller particle size and inhibitory effects on hematopoiesis.

Silkworm fed with 100 ppm of Ag NPs increased production levels of ROS, which resulted in cell apoptosis, necrosis, and DNA damage [132]. This mortality rate was higher when compared to silkworm groups fed with 1 and 10 ppm of Ag NPs. These results are in accordance with studies carried out by Meng et al., who stated that although concentrations of Ag NPs greater than 800 mg/L improved the growth rate of silkworms, it also resulted in silkworm death [122]. Similar reports indicated that although increasing concentrations of Ag NPs positively reflected on the bodyweight of the silkworm, the exposure to Ag NPs caused adverse effects on tissues and might have induced harmful effects on primary organs [133]. Chen et al. [134] studied the toxic effects of different concentrations (100, 200, and 400 mg/L) of Ag NPs on the midgut tissues of the silkworm. It was reported that increasing concentrations of Ag NPs led to the development of poisoning symptoms and caused damages to the tissues of the silkworms exposed to 400 mg/L of Ag NPs, affecting the silkworm midgut. It was also shown that increasing concentrations of Ag NPs resulted in a downregulated expression of digestive enzymes which caused damages to the silkworm tissues and suppressed the activity of the enzyme SOD and of the protein HSP 1. This, in turn, activated the oxidative stress pathway and the production of ROS, which have toxic effects on the silkworm digestive system.

### Effects of nanomaterial exposure on silkworm tissues and organs

The application of nanotechnology in some aspects of science is still new and the evaluation of its effects on tissues and organs of model organisms is important. The silk gland is present mostly in the larval stage of the silkworm [135] and it is responsible for storing and synthesizing silk proteins (i.e., sericin and fibroin) [136]. Chemical residues from the application of fungicides and pesticides to crops may contaminate mulberry leaves [137] and lead to damage or death of important tissues and organs when ingested by the silkworm. Li et al. [137] studied the effects of feeding TiO_2_ NPs as a treatment to phoxim-exposed silkworms. The silk gland of the silkworm larvae fed with mulberry leaves containing phoxim residue was damaged, showing large vacuolization in the gland lumen and epithelial cell sparseness [137]. Feeding the silkworm with TiO_2_ NPs was reported to reverse the damage caused by phoxim exposure. It was also shown that phoxim exposure may result in a decrease in fibroin concentration and in sericin genes (ser2 and ser3), which explains the vacuolation in the silk gland and the reduced cocooning rate [137].

Innate immunity is the first line of defense mechanism against pathogens, preventing bacterial and viral infections [138] due to the phagocytosis of pathogens and dead cells [139]. Hemocytes are blood cells of invertebrates that are used as models to study the effects of drugs and nanomaterials on the innate immune system and on DNA damage mechanisms. The fast replication of hemocytes in invertebrates is documented as an advantage [140] when compared to mammalian models. Rajasekharreddy et al. [141] focused on the effect of flavonoids (FLV), biosynthesized flavonoid Ag NPs (FLV-Ag NPs), and Ag NPs on silkworms infected with *Staphylococcus aureus*. They explained that by giving FLV and FLV-Ag NPs to these silkworms their hemocyte density was increased, which accounted for their bactericidal activity against *S. aureus*. Additionally, bacterial growth was significantly higher in control groups in comparison to groups treated with biosynthesized FLV-Ag NPs, which inhibited bacterial growth.

Ag NPs are known for their attractive physicochemical properties and their antimicrobial applications [142–143], and research reports have indicated that Ag NPs are lethal to bacteria and other pathogens [142–145]. Aside from the antimicrobial applications of Ag NPs, Govindaraju et al. [146] reported that an antiviral assay using Ag NPs synthesized with *Spirulina platensis* was efficient in controlling a *Bombyx mori* nuclear polyhedrosis virus (BmNPV) infection by increasing hemocyte density in the silkworm. An increased hemocyte population in invertebrates indicates that there is a defense mechanism taking place in order to deal with foreign cells. Quantum dots have unique optical properties that are used in biological imaging [147–150]. They are also known for their size-dependent cytotoxicity [151–152]. Silkworms were subjected to doses of 32 mM of CdTe QDs, 1 µg/µL of citric acid–nitrogen-doped carbon dots (C-NCDs), and to 0.39 µg/µL of Si NPs in order to study the differences in the immune responses and programmed cell death induced in hemocytes [153]. It was shown that autophagy and apoptosis caused by Si NPs were reverted and experimental groups exposed to C-NCDs, CdTe QDs caused autophagy, apoptosis, and necrosis in the hemocytes.

Xing et al. [154] studied the outcome of introducing Si NPs in the hemolymph of the silkworm. It was reported that 3.9 µg of Si NPs was toxic to the hemocytes when compared to the groups exposed to 0.39 and 0.039 µg of Si NPs. A high dose of Si NPs (3.9 µg) did not increase the production of ROS and it was suggested that the its effect on hematopoiesis was self-repaired and the damage to the hematopoietic tissues was limited. Zou et al. [155] studied the efficacy in using silver nanoparticle colloids (AgNPC) to reduce the infection caused by BmNPV in silkworms. It was shown that AgNPC were able to improve the survival rate of the silkworm from 22 to 67% when compared to the control untreated group. Adherence of the virus to AgNps led to the production of free radicals which penetrated and disintegrated the virus capsids, proteins, and DNA of BmNPVs. This decreased the virus pathogenicity and improved the survival rate of the affected silkworms.

### The effects of nanomaterials on the silk fibers of silkworms

Silkworm silk is gaining popularity in the textile industry because of its shiny appearance and excellent mechanical properties [156–158]. Improving the luster and the quality of silkworm silk is imperative and studies are being carried out in this direction. Feeding silkworms with nanomaterials, such as carbon nanotubes (CNTs), titanium dioxide, copper, and graphene has been reported to improve the mechanical properties and secondary structures of silkworm silk [159–160].

To study silk quality, the mechanical properties, crystalline structure, and thermal stability of the silk fiber are usually taken into consideration. Scientists have reported that the hindrance of the conformational transition of silk fibroin from a random coil/α-helix to β-sheet contributes to increased breaking elongation and toughness modules, which translates as excellent mechanical properties [161].

Studies focusing on the impacts of nanomaterials on silkworms and on their silk fibers have been published recently. Cai et al. [161] focused on producing intrinsically modified silk fibers via feeding silkworms with a TiO_2_ artificial diet at different concentrations (1, 2, 3, and 4%). It was reported that feeding silkworms with a 1% TiO_2_ artificial diet significantly improved the mechanical properties and ultraviolet resistance of the silk fiber when compared to control (mulberry feed). Increasing concentrations of the TiO_2_ artificial diet reduced breaking strength and ultraviolet resistance. It was also shown that 1% of TiO_2_ NPs confined the conformation transition of silk fibroin from a random coil/α-helix to β-sheet, which translates into a more evident confined crystallization effect.

Single-walled nanotubes (SWNTs) and graphene limited the conformation transition of silk fibroin from a random coil/α-helix to β-sheet, which means that the modified diet of the silkworm positively affected the secondary structure of the silk fiber and the graphitic structure enhanced its electrical conductivity [162]. Graphene has excellent electrical conductivity, optical properties, and mechanical strength [163–166], and these traits were conferred to the silk fibers. It was also indicated that silkworms fed with a lower concentration (0.2 wt %) of SWNTs exhibited improved mechanical properties of their fibers when compared to silkworms fed with 2 wt % of graphene, which exhibited the least improved silk mechanical properties. Cheng et al. [167] demonstrated that Cu or Ag NPs introduced into the silkworm larval diet were incorporated into the silk fibers, enhancing their mechanical properties and promoting silk protein crystallization. This is in line with the research report by Wu et al*.* [168], who stated that silkworm silk containing Cu exhibited a good tensile strength of 360 MPa and a strain of 38%, which is 89% and 36% higher than the values of tensile strength and strain obtained for natural silk fiber (control), respectively. Ma et al*.* [169] evaluated the effect of vascular injection of graphene quantum dots (GQDs) in silkworm on the mechanical properties of the silk. GQDs are known for their outstanding mechanical properties and for their application as a reinforcement material [163]. It was reported that 0.6 μg of GQDs absorbed by the silkworm resulted in silk fibers with an improved crystallization, which may be the reason for the improvement of the mechanical properties of the silk fibers.

**Figure 2:**
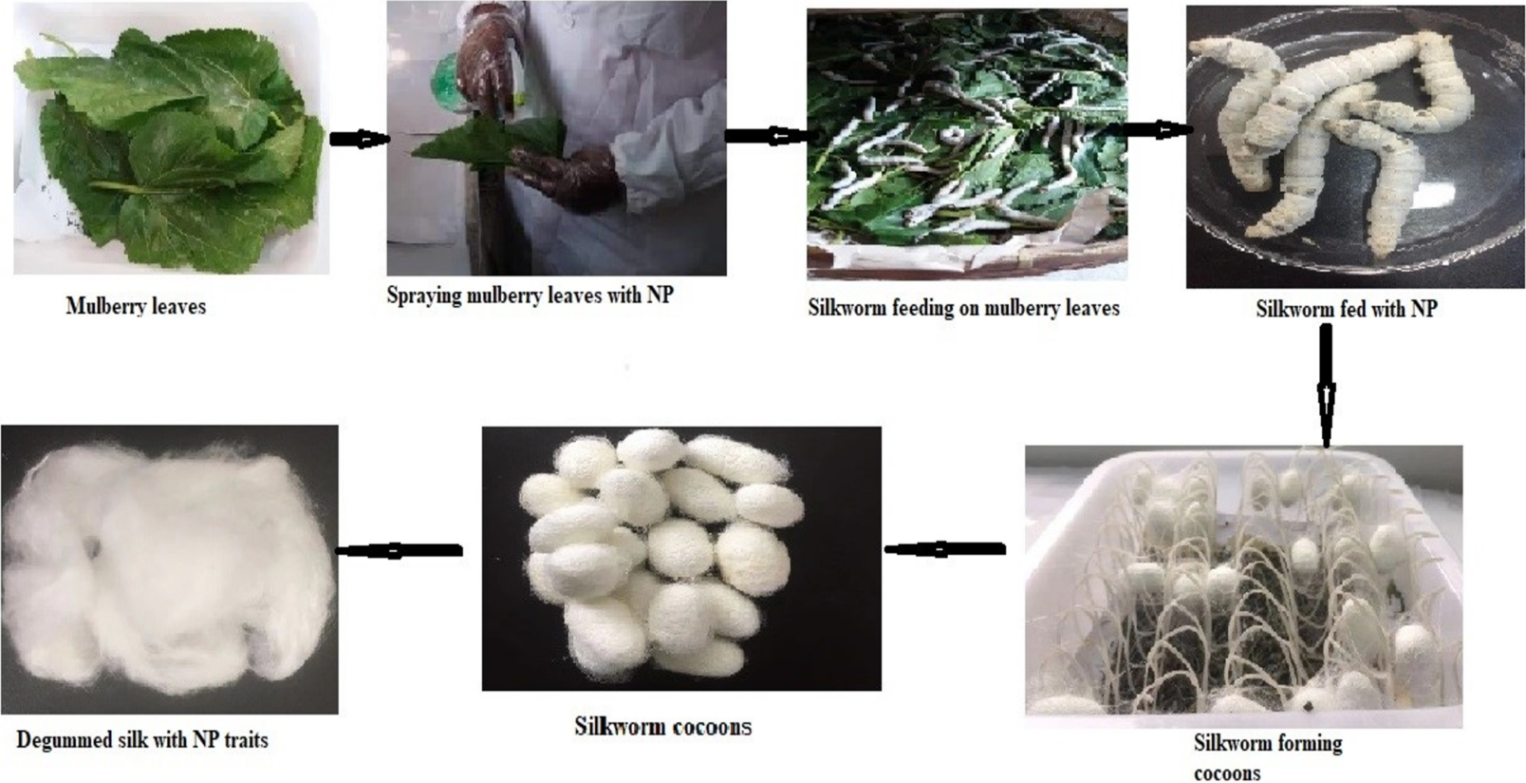
An illustration of how nanomaterials are directly fed to the silkworm. ©Sandra Senyo Fometu.

Xu et al. reported that feeding silkworm larvae with unpurified composites of CNTs and lignosulfonate composite (LGS) resulted in excess LGS coating, which blocked CNTs from being embedded into the silk fiber. The purification of CNT/LGS composites resulted in a higher CNT content, which led to an ordered graphitic structure in the resulting silk fiber with a mechanical strength of 1.07 GPa and a strain of 16.8% [170]. The effects of bovine serum albumin (BSA)-stabilized gold nanoclusters (BSA-Au NCs) at different concentrations (9.38, 1.88, 0.938, and 0.188 µg) inserted via intravascular injection in the silkworm was studied by Ma et al*.* [171]. It was shown that silkworms injected with 9.38 µg exhibited enhanced silk mechanical properties with elongation at break, breaking strength, and toughness modulus of 38.02%, 368.88 MPa, and 104.12 MPa, respectively, when compared with the control group (36.79%, 292.66 MPa, and 78.79 MPa, respectively). The crystalline structures of BSA-Au NCs silk were unchanged with an enhanced crystallization of the silk proteins. Zhang et al. [172] focused on the impact of glucose-coated Ag NPs on the silkworm larvae diet and on silk protein synthesis. Silk fibers exposed to glucose-coated Ag NPs exhibited a smooth surface with significant changes in the silk diameter. Ag NPs concentrations of 0.20% and 0.02% were reported to promote the synthesis of silk proteins, improve the mechanical properties of the silk fibers, and to improve the antibacterial properties of the silk. Figure 2 illustrates how nanoparticles are used in the feeding of the silkworm larvae, how the cocoons are formed, and how degummed silk fibers with NP traits look like.

## Conclusion

This paper highlighted reports on the application of nanomaterials in sericulture with a key focus on the effects on the survival rate, silk fiber quality, and on the growth and development of the silkworm. The introduction of nanomaterials through diet or injections has been reported to improve quality, tissue repair, and the overall survival rate of the silkworm. Despite the positive reports of nanomaterials on silkworms, high doses of nanomaterials, such as Ag NPs, CdTe QDs, and Si NPs have shown to induce an excessive production of ROS, which causes oxidative stress leading to cell apoptosis and autophagy. This review also discussed the effects of nanomaterials on the silk fibers. Reports indicate that the presence of these nanomaterials did not damage the crystalline structure of the silk and it also contributed to an increased elongation and toughness of the silk fiber. In addition, the exposure of diseased silkworms to some nanomaterials also exhibited some therapeutic properties. For instance, feeding silkworms with TiO_2_ NPs was reported to reverse the damage caused to the silk gland due to phoxim exposure. Also, Ag NPs improved the overall survival rate of silkworms infected with BmNPV.

The toxicity of nanomaterials to the ecosystem is a major concern and it is imperative to evaluate their effects. Therefore, nanomaterials with varied sizes and concentrations need to be employed in experimental trials using silkworms as an animal model to extend and improve research reports and literature in science. Also, more nanomaterials need to be exploited to investigate the recovery effects following an exposure of diseased silkworms to nanoparticles in order to understand the interaction between NPs and silkworms.
